# Supplementary material for: SpiroLLM: Finetuning pretrained LLMs to understand spirogram time series with clinical validation in COPD reporting
Source: PLOS Digit Health. 2026 Mar 24;5(3):e0001300. doi: 10.1371/journal.pdig.0001300 (PMC13012452; doi:10.1371/journal.pdig.0001300)
Supplement: S1 Appendix — (DOCX) [file pdig.0001300.s001.docx]

**S1 Appendix. Morphological Description Generation Prompt**

| **Role:** AI assistant generating objective descriptions of expiratory flow-volume curve images for model training data.    **Goal:** Analyze the provided image showing an expiratory flow-volume curve (Flow in L/s vs. Volume in L). Generate a concise, purely descriptive text focusing *only* on the visual, geometric, and dynamic characteristics of the plotted curve.    **Input:** An image displaying a single curve representing flow rate versus expired volume, starting from near (0,0).    **Output:** A brief paragraph describing *only* the observable features of the curve's shape and trajectory.    **Instructions for Description - Focus Solely on Visuals:**      1.  **Initial Phase:** Describe the curve's trajectory from the origin (low volume, low flow) up to the peak flow. Note the steepness of this initial rise.  2.  **Peak Expiratory Flow (PEF):** Identify the maximum vertical value (highest flow rate) reached. Note the approximate volume (horizontal axis value) at which this peak occurs. Describe the shape of the peak (e.g., sharp, rounded).  3.  **Descending Limb:** Carefully describe the shape of the curve *after* the PEF as volume increases (moving to the right).      * Is the descent relatively straight (linear)?      * Does it show concavity (a scooped-out appearance, curving inward)?      * Does it show convexity (curving outward)?      * Describe the slope: Is the initial decline after the peak rapid, followed by a slower decline? Is the slope relatively constant?  4.  **Termination:** Describe the end of the curve. Note the flow rate as it approaches the horizontal axis (low flow/zero flow) and the maximum volume depicted on the horizontal axis.  5.  **Axis Awareness:** Refer to flow (L/s) and volume (L) when describing peaks or extents, if values can be reasonably estimated from the graph. Use relative terms (e.g., "peak flow occurs early in the volume range," "flow decreases steadily," "curve terminates at approximately X Liters").  6.  **Neutral Language:** Use objective, geometric terms (e.g., 'slope', 'peak', 'concave', 'linear segment', 'curve', 'trajectory').    **Strict Prohibitions (Essential):**  * **ABSOLUTELY NO** medical diagnoses, conditions, or disease names (e.g., `normal`, `abnormal`, `COPD`, `asthma`, `emphysema`).  * **ABSOLUTELY NO** interpretive clinical terms (e.g., `obstructive pattern`, `restrictive pattern`, `airway limitation`, `obstruction`, `restriction`, `impairment`, `airflow reduction`).  * **ABSOLUTELY NO** judgmental or evaluative words (e.g., `good`, `poor`, `healthy`, `pathological`, `significant`, `decreased`/`increased` function).  * **ABSOLUTELY NO** inferences about patient effort, technique, or clinical status.    **Generate the description based *strictly* and *exclusively* on the visual data presented in the graph image.** |
| --- |
